# Targeting minimal residual disease (MRD) in colorectal cancer: Therapeutic strategies, clinical trials, and the path toward molecular cure

**DOI:** 10.1016/j.cpt.2026.07.003

**Published:** 2026-07-23

**Authors:** Yusuf Saleem Khan, Aisha Farhana, Hatim Adam Nagi, Alfatih Mohamed Ahmed Alnajib, Tarig Ginawi, Abuzar Abdulwahab Osman, Abdullah Alsrhani, Emad Manni

**Affiliations:** a Department of Anatomy, College of Medicine, University of Ha'il, Ha'il 55473, Saudi Arabia; b Department of Clinical Laboratory Sciences, College of Applied Medical Sciences, Jouf University, Sakaka, Aljouf 72388, Saudi Arabia; c Department of Family and Community Medicine, College of Medicine, University of Ha'il, Ha'il 55473, Saudi Arabia; d Department of Surgery, College of Medicine, University of Ha'il, Ha'il 55473, Saudi Arabia; e Department of Biochemistry, College of Medicine, University of Ha'il, Ha'il 55473, Saudi Arabia; f Department of Pharmacology, College of Medicine, University of Ha'il, Ha'il 55473, Saudi Arabia

**Keywords:** Minimal residual disease (MRD), Circulating tumor DNA (ctDNA), Cell-free nucleic acid, Colorectal neoplasm, Liquid biopsy, Adjuvant chemotherapy, Biomarkers, Surrogate endpoint, Immunotherapy, Clinical trials

## Abstract

The management of early-stage colorectal cancer (CRC) is undergoing a significant transformation, from traditional clinical and pathological staging to a dynamic, molecular signature-driven approach utilizing circulating tumor DNA (ctDNA). Postoperative detection of minimal residual disease (MRD) has emerged as a strong independent prognostic biomarker. It identifies patients at high risk of recurrence while reducing unnecessary exposure to adjuvant chemotherapy for those cured by surgery alone. However, this success reveals a critical therapeutic dilemma. Standard adjuvant chemotherapy has a limited ability to eliminate MRD. Consequently, the molecularly defined high-risk subgroup currently has no proven therapy that effectively eradicates MRD and improves survival. In this review, we synthesize emerging therapeutic strategies designed to address this unmet need. We discuss ongoing clinical trials investigating intensified cytotoxic regimens, MRD-guided targeted therapies, and novel immunotherapy combinations, especially for microsatellite-stable (MSS) disease. Furthermore, this review examines the role of ctDNA dynamics as a surrogate endpoint for regulatory drug approval, a crucial step in accelerating the development of these novel therapies. Finally, we address the real-world challenges of implementation, including access disparities, equity gaps, and standardized clinical algorithms. We highlight how emerging technologies and global collaborations may further refine MRD detection. The eradication of MRD represents the next frontier in curing CRC; however, this demands focused clinical research and validated surrogate endpoints to translate prognostic insights into tangible survival benefits.

## Highlights

• Circulating tumor DNA (ctDNA)-based minimal residual disease (MRD) detection is a strong independent prognostic indicator of recurrence in early-stage colorectal cancer.
• Conventional adjuvant chemotherapy has limited efficacy in eradicatingeradicate MRD in the majority of ctDNA-positive patients.
• Novel approaches include cytotoxic intensification, targeted therapy, and immunotherapy.
• ctDNA clearance is being validated as a surrogate to accelerate regulatory approval.
• Implementation requires test standardization, equitable access, and evidence-based clinical algorithms.

## Graphical abstract

This graphical abstract illustrates the multifaceted approach required to translate MRD detection into improved clinical outcomes for colorectal cancer. The central theme, "Bridging MRD detection in CRC to clinical cure," connects four interconnected domains: (1) mechanisms of MRD persistence: cancer stem cell dormancy, immunosuppressive niche, clonal evolution, and metabolic adaptation; (2) novel therapeutic strategies: cytotoxic intensification, MRD-guided targeted therapy, and immunotherapy combinations for MSS tumors; (3) regulatory roadmap: validating ctDNA as a surrogate endpoint through trial- level validation, standardization, and real-world evidence; and (4) implementation framework: ensuring equitable access, cost- effectiveness, and standardized clinical algorithms. Together, these components illustrate the comprehensive strategy needed to translate the prognostic power of ctDNA into curative therapeutic paradigms for MRD-positive colorectal cancer. Abbreviations: CRC: Colorectal cancer; ctDNA: Circulating tumor DNA; MRD: Minimal residual disease; MSS: Microsatellite-stable.

**Figure:**
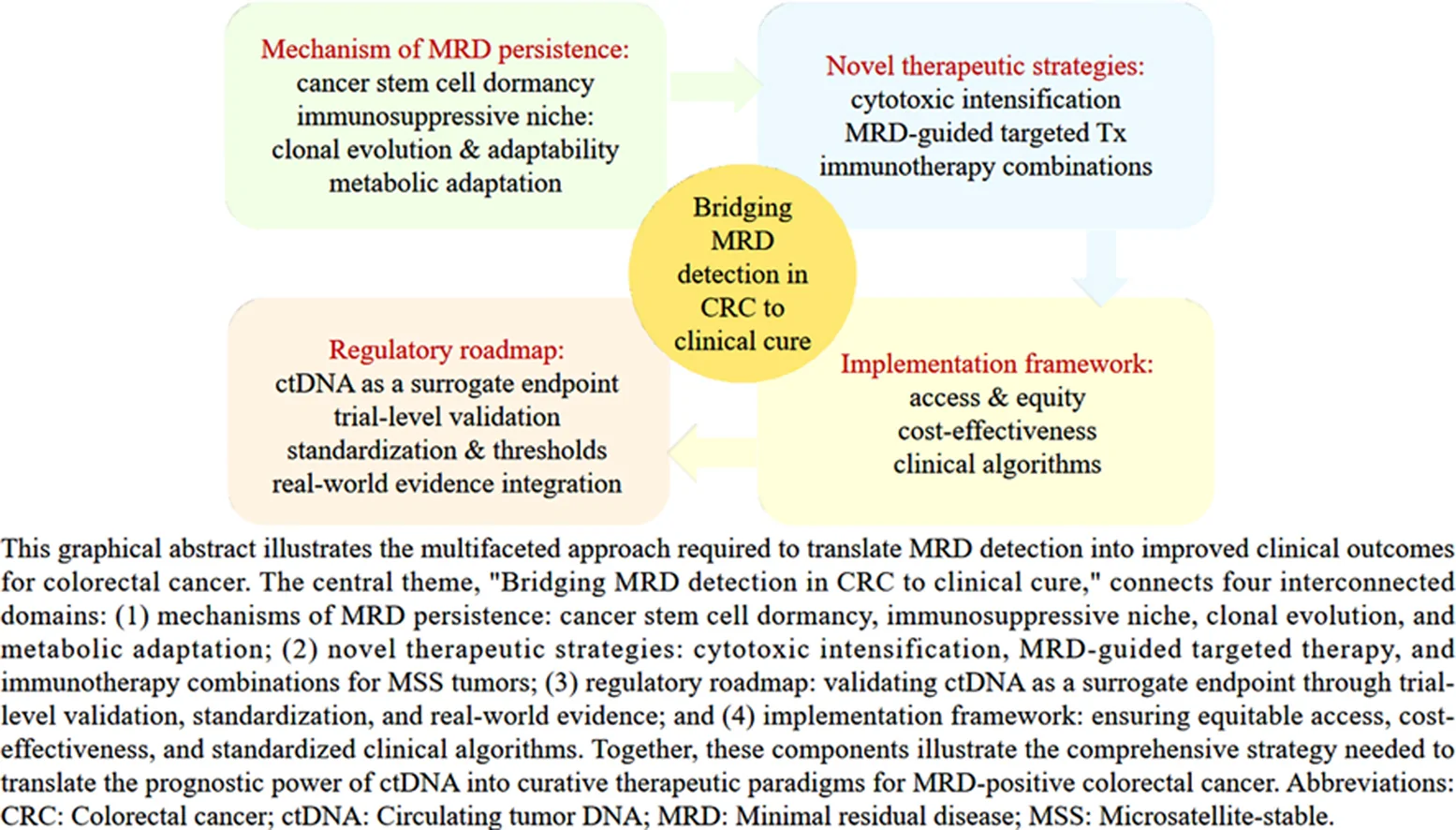

## Introduction

Colorectal cancer (CRC) remains the third most commonly diagnosed malignancy and a leading cause of cancer-related mortality worldwide.^1^ A critical determinant of patient survival is the prevention of disease recurrence after curative-intent surgery for localized stages II/III.^2^ However, recurrence remains a major clinical challenge, driven primarily by residual micrometastatic disease undetectable by conventional imaging. This occult disease poses the primary threat to long-term survival. Clinical and pathological staging have traditionally guided risk stratification and decisions regarding adjuvant chemotherapy following surgical resection.^3^^,^^4^ However, this approach has well-documented limitations, including the risk of overtreating patients who are already cured and potentially undertreating those with residual disease. This inability to accurately identify patients with minimal residual disease (MRD) remains a fundamental challenge in oncology. The emergence of liquid biopsy, particularly circulating tumor DNA (ctDNA), is fundamentally transforming this paradigm.

Liquid biopsy using ctDNA has introduced a transformative era for cancer management. This technology is based on the principle that apoptotic or necrotic and, to a lesser extent, actively secreting tumor cells release DNA fragments into the circulation, providing a real-time, dynamic snapshot of the tumor genome.^5^ The development of highly sensitive detection methods now enables precise identification of MRD. Two primary technological approaches have been validated: tumor-informed assays and tumor-agnostic (plasma-only) assays. First are the tumor-informed assays, which use sequencing of the resected tumor to design a patient-specific panel that tracks known mutations in plasma. This approach offers high sensitivity and specificity for MRD detection.^6^ In contrast, the tumor-agnostic or plasma-only assays analyze cell-free DNA using multimodal strategies without prior tumor sequencing. These assays have demonstrated robust clinical performance and improved the accessibility of MRD testing.^6^^,^^7^

The tumor-informed approach is widely regarded as the leading strategy for MRD detection in the adjuvant or curative-intent setting because of high sensitivity and specificity.^7^^,^^8^ This method involves sequencing resected tumor tissue to identify clonal, single-nucleotide variants. These variants are then used to design a personalized panel for tracking mutations in plasma cell-free DNA.^8^ To improve detection at ultra-low allele frequencies, the field is evolving toward broader sequencing strategies. While early tumor-informed assays relied on targeted panels covering hundreds of genes, newer approaches use whole-exome sequencing (WES) or whole-genome sequencing (WGS) to capture a larger repertoire of patient-specific somatic variants.^9^ WGS-based assays offer additional advantages by analyzing both coding and non-coding regions, enabling the selection of hundreds of clonal variants per patient.^9^^,^^10^ This broader variant coverage increases the probability of detecting ctDNA at very low allele frequencies (< 0.01%) and demonstrates higher sensitivity than target panel-based approaches in CRC.^10^^,^^11^

The phase II/III CLAUDIA platform trial (KCSG CO22-12; NCT05534087) is a multicenter platform study that represents an important methodological advancement by employing a WES-informed ctDNA assay capable of tracking up to 100 patient-specific somatic variants. This ultra-deep, personalized approach improves detection sensitivity and enables precise detection of MRD at very low variant allele frequencies. 12, 13, 14 Importantly, the trial uses ctDNA detection to identify patients who may benefit from intensified adjuvant chemotherapy. This design exemplifies the next generation of MRD-directed trials, where advanced diagnostics directly influence therapeutic decisions. Tumor-informed assays offer high positive predictive values and low false-positive rates, making them well-suited for precise prognostic stratification.^15^ They also support clinical decisions by providing detailed molecular information on treatment options and expected outcomes. However, these advantages are tempered by practical limitations, including longer turnaround time and higher cost, as both tumor tissues and blood samples are required.

In contrast, tumor-agnostic or plasma-only assays provide a more streamlined and accessible alternative. These assays use fixed panels to analyze predefined genomic or epigenomic alterations directly from plasma, eliminating the need for prior tumor sequencing.^16^ This design provides notable practical advantages, including faster turnaround times and lower costs. However, this convenience may come at the expense of analytical sensitivity. These assays are generally less effective at detecting very low ctDNA levels seen in MRD.^17^ Consequently, although their role in MRD detection is still evolving, they are currently more suitable for applications in advanced-stage disease. A recent meta-analysis supports this distinction, showing that tumor-informed assays achieve superior sensitivity and specificity in MRD settings, particularly at low ctDNA levels.^18^ In contrast, tumor-agnostic assays offer greater accessibility and speed but may have reduced sensitivity in low-shedding disease.

A rapidly emerging class of liquid biopsy assays focuses on detecting cancer-specific DNA methylation patterns. These assays assess epigenetic changes, specifically methylation at cytosine-phosphate-guanine (CpG) sites, which are often altered in a highly tissue-specific manner in cancer cells.^19^^,^^20^ This approach offers several advantages. First, the abundance of methylation markers can generate a robust detection signal, potentially improving sensitivity. Second, the tissue-specific nature of DNA methylation patterns enables not only ctDNA detection but also identification of the tumor's tissue of origin.^21^ This combined capability is not present in mutation-based assays.^22^ Although methylation-based assays are less established in MRD detection, several are in advanced development and undergoing clinical evaluation, with promising results.

The prognostic value of postoperative ctDNA in CRC is well established. Large prospective studies show that ctDNA is a strong predictor of recurrence in stage II-III disease, outperforming traditional clinicopathological risk factors.^23^, ^24^, ^25^ The DYNAMIC trial (ACTRN12615000381583) demonstrated that ctDNA-guided management safely reduces the use of adjuvant chemotherapy in stage II colon cancer.^26^^,^^27^

Despite this prognostic success, a critical therapeutic gap remains. Standard adjuvant chemotherapy fails to prevent recurrence in most ctDNA-positive patients, highlighting the need for effective MRD-directed therapies.^28^^,^^29^ This review synthesizes current evidence, with a focus on emerging clinical trials targeting MRD-positive CRC. We also examine efforts to validate ctDNA clearance as a surrogate endpoint, outline the current landscape of active trials, and discuss key challenges and opportunities in this rapidly evolving field.

To ensure a comprehensive and balanced review, the relevant literature was identified through searches of PubMed/MEDLINE, Scopus, Web of Science, and Google Scholar using combinations of keywords including CRC, MRD, ctDNA, tumor-informed assay, mismatch repair deficiency (dMMR), clinical trials, clonal hematopoiesis, epigenetics, tumor microenvironment, liquid biopsy, immunotherapy, artificial intelligence, and precision oncology. Emphasis was placed on peer-reviewed publications from 2020 to 2026 to capture recent advances in molecular mechanisms, diagnostics, and therapeutic strategies. Seminal and foundational studies were intentionally included where they established key biological concepts, mechanistic insights, methodological advances, or landmark clinical evidence, including pivotal clinical trials and consensus guidelines, that continue to inform current research and clinical practice. Additional relevant publications were identified through manual screening of reference lists from selected articles. The selected literature was critically evaluated to integrate mechanistic, translational, and clinical evidence relevant to host microRNA dysregulation in virus-associated cancers.

## The biology of minimal residual disease (MRD) and therapeutic resistance

The clinical challenge of eradicating MRD is driven by fundamental biological properties that confer resistance to conventional therapies. Understanding these processes is essential for developing effective treatment strategies [Figure 1]. Persistent ctDNA after curative-intent therapy reflects the presence of viable residual tumor cells with distinct survival advantages, rather than a passive phenomenon.^5^^,^^29^ Several interconnected mechanisms contribute to this persistence. One key mechanism involves chemoresistant, dormant stem cells. These cells are not defined by a single marker but by a conserved molecular program and often exhibit activation of developmental pathways such as Wnt/β-catenin and Notch, which support self-renewal and maintain a quiescent, non-proliferative state. This phenomenon is associated with specific surface markers, such as leucine-rich repeat-containing G-protein-coupled receptor 5 *(LGR5)*, a known marker of intestinal stem cells. By entering this dormant state, cancer stem cells (CSCs) effectively evade cell-cycle-dependent cytotoxic agents such as 5-FU and oxaliplatin, allowing them to persist and drive late recurrence.^30^^,^^31^ This biology provides a sound rationale for cytotoxic intensification strategies, such as multi-agent regimens like FOLFIRINOX (folinic acid [leucovorin], fluorouracil [5-FU], irinotecan, and oxaliplatin), which aim to target heterogeneous cell populations and overcome resistance.

**Figure 1:**
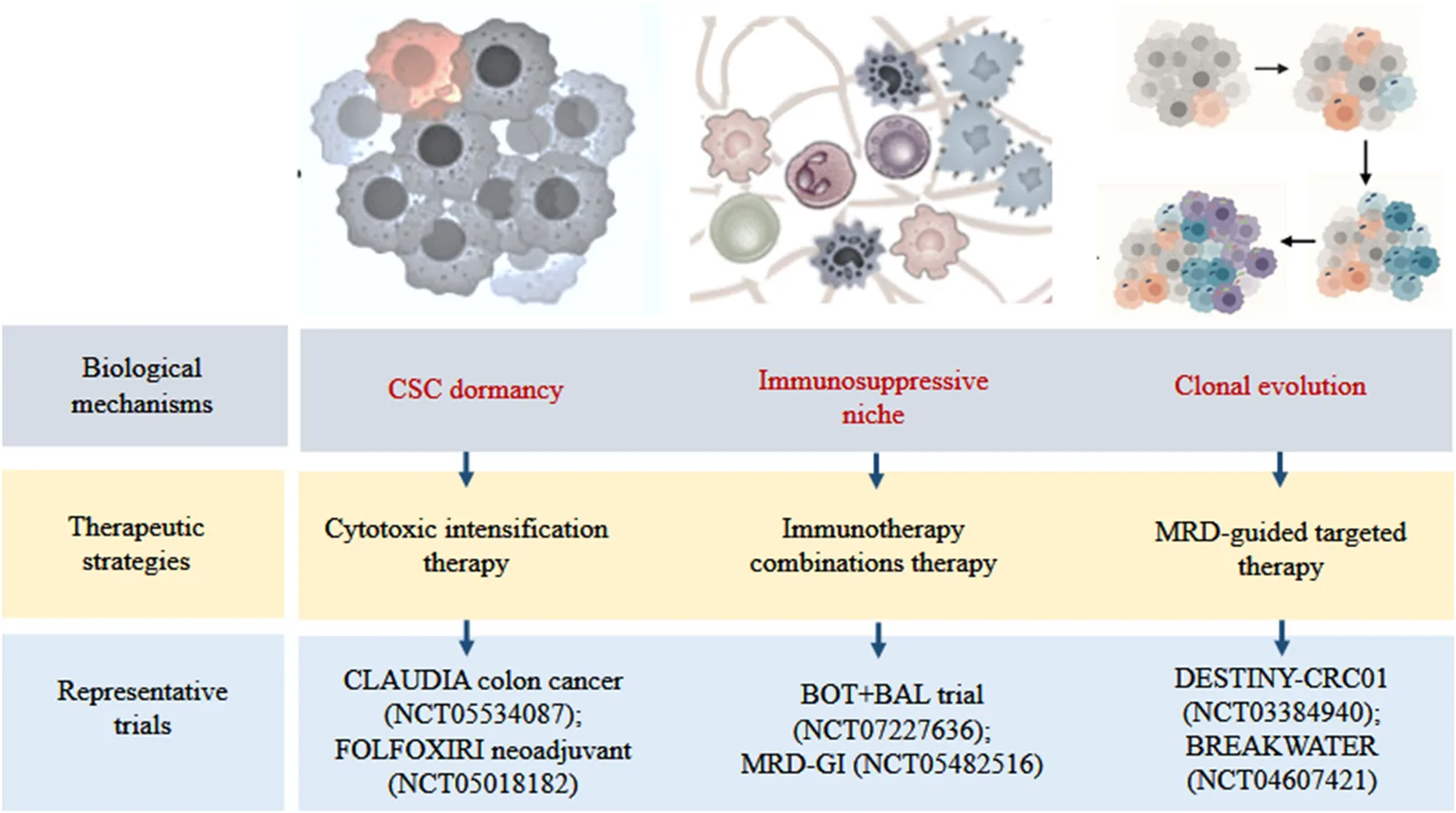
Biological mechanisms underlying MRD persistence and corresponding therapeutic strategies. Approaches: CSC dormancy, in which quiescent CSCs evade conventional chemotherapy, driving the investigation of cytotoxic intensification regimens (e.g., the modified FOLFOXIRI [NCT05018182]) to eradicate these resistant cells. Immunosuppressive niche, where the protective tumor microenvironment, comprising Tregs and MDSCs, shields residual cells. This niche can be targeted through immunotherapy combinations like the BOT + BAL Trial [NCT07227636] to reactivate antitumor immunity. Clonal evolution, in which therapy-resistant subclones emerge under selection pressure, supports the use of MRD-guided targeted therapy (e.g., the DESTINY-CRC01 trial [NCT03384940] in CRC) to precisely eliminate resistant populations. Additional representative trials, such as the CLAUDIA colon cancer study (NCT05534087), further illustrate these investigative strategies. CRC: Colorectal cancer; CSC: Cancer stem cell; GI: Gastrointestinal; MDSC: Myeloid-derived suppressor cell; mFOLFOX6: Modified folinic acid + fluorouracil + oxaliplatin; MRD: Minimal residual disease; PD-1: Programmed cell death protein 1; Treg: Regulatory T cell.

Another major contributor to MRD persistence is the establishment of an immunosuppressive micrometastatic niche. Residual tumor cells actively shape this microenvironment by recruiting host immune cells and producing inhibitory signals. The niche is enriched with regulatory T-cells (Tregs), myeloid-derived suppressor cells (MDSCs), and potent inhibitory cytokines such as transforming growth factor-beta (TGF-β) and interleukin-10 (IL-10).^32^ These cytokines directly suppress cytotoxic T-cell and natural killer (NK) cell functions. In addition, they promote the upregulation of multiple immune checkpoint molecules, including lymphocyte-activation gene 3 (*LAG-3*) and T-cell immunoglobulin and mucin-domain containing-3 (*TIM-3*), contributing to T-cell exhaustion and dysfunction. This multi-faceted immunosuppressive network shields MRD from immune surveillance and may partly explain the limited efficacy of single-agent immune checkpoint inhibitors in microsatellite-stable (MSS) CRC.^33^ These insights support the investigation of combination immunotherapy strategies. Such approaches aim to simultaneously activate antitumor immunity and disrupt the protective tumor microenvironment, offering a more effective strategy for eliminating MRD.

The process of clonal evolution under therapeutic pressure is a key driver of MRD persistence, shaping the residual disease by selecting for clones with intrinsic resistance or new targetable alterations [Figure 1]. This concept underpins MRD-guided integration of novel targeted agents. In this approach, ctDNA serves as a dynamic biomarker to identify persistent and often aggressive subclones.^34^ This enables precision treatments with agents such as B-Raf proto-oncogene, serine/threonine kinase *(BRAF)* or *HER*2 inhibitors. While these therapies may have limited impact on the primary tumor, they may be critical for eliminating the resistant residual disease that drives recurrence. This approach reflects a paradigm shift in cancer treatment. The goal is to target the specific biological mechanisms that sustain residual disease and to intervene at points of greatest vulnerability [Figure 1].

3. Absence of a standard of care for minimal residual disease (MRD)-positive patients

The robust validation of ctDNA as a prognostic biomarker has created a new, molecularly defined high-risk patient group. This group includes patients with detectable MRD following curative-intent surgery. However, this advance presents a major therapeutic dilemma. Clinicians can now identify patients at high risk of recurrence, yet there is currently no universally accepted standard-of-care therapy that has been shown to reliably clear MRD and prevent recurrence to manage this risk.

### The limits of conventional chemotherapy

Adjuvant treatment for stage III and high-risk stage II CRC has traditionally relied on fluoropyrimidine-based chemotherapy, usually in combination with oxaliplatin.^35^ However, multiple ctDNA-based MRD studies have demonstrated that a large proportion of patients still harbor residual disease despite standard therapy. Persistent ctDNA positivity strongly predicts recurrence.^36^ The same clinical trials that established ctDNA as a well-validated prognostic biomarker have revealed the limitations of current chemotherapy in effectively eradicating MRD. Subgroup analyses from key studies provide consistent evidence. In the DYNAMIC trial for stage II colon cancer, patients who remained ctDNA-positive after surgery and subsequently received adjuvant chemotherapy (most commonly capecitabine plus oxaliplatin [CAPOX] or folinic acid, fluorouracil, and oxaliplatin [FOLFOX]) still had a significantly higher risk of recurrence than ctDNA-negative patients.^26^ These findings indicate that standard chemotherapy does not fully reverse the adverse prognosis associated with persistent ctDNA. This observation is further corroborated by larger, real-world cohort studies. Data from the CIRCULATE program and other international cohorts have consistently shown that postoperative ctDNA positivity is associated with high recurrence rates within the first few years after surgery, despite standard adjuvant treatment.^37^ Importantly, chemotherapy appears to reduce this risk only marginally. However, the majority of ctDNA-positive patients still experience disease recurrence. Recent data from the DYNAMIC-III trial (NCT04486378) extend these observations to stage III disease.^38^ Unlike earlier studies focused primarily on treatment de-escalation, DYNAMIC-III evaluates both escalation and de-escalation based on postoperative ctDNA status. Early findings suggest that ctDNA-guided approaches improve risk stratification beyond conventional staging and may help identify ctDNA-negative patients with stage III disease who could safely avoid or de-escalate adjuvant chemotherapy, as well as ctDNA-positive patients who have a poor prognosis despite standard treatment.^38^ Additional prospective studies are now evaluating strategies to optimize adjuvant chemotherapy using ctDNA guidance. The REVISE trial (NCT06242418), a phase II multicenter randomized study, is investigating whether serial ctDNA monitoring can enable dynamic adjustment of chemotherapy regimens in stage III colon cancer. This approach aims to improve treatment efficacy by identifying patients who require escalation while minimizing unnecessary toxicity in those who do not benefit.^39^ However, the continued risk of recurrence in ctDNA-positive patients, even with treatment intensification, highlights a key limitation: escalating cytotoxic therapy alone may be insufficient to overcome the aggressive biology of MRD-positive disease. Together, these findings underscore a significant unmet clinical need. Current adjuvant chemotherapy regimens are insufficient for many MRD-positive patients, emphasizing the need for more effective biology-driven therapeutic strategies.

### The imperative for clinical trial enrollment

In the absence of a proven standard of care that alters the natural history of MRD-positive disease, a clear consensus has emerged among oncologists and professional societies: patients with detectable ctDNA after curative-intent therapy should be prioritized for clinical trial enrollment.^40^ This reflects a fundamental principle in oncology when managing a high-risk disease without a reliably effective treatment option. Although standard chemotherapy is often used in this setting, it is widely recognized as suboptimal and associated with a high probability of failure. Therefore, the most rational and ethically appropriate strategy is to enroll these patients in well-designed clinical trials aimed at identifying interventions that can eradicate MRD and prevent recurrence.^41^ However, the complexity of these investigations is highlighted by recent trial experience. The phase II component of NRG-GI005 (COBRA) trial (NCT04068103) was terminated early following a prespecified futility analysis after ctDNA-guided adjuvant chemotherapy failed to demonstrate improved ctDNA clearance compared with surveillance. Specifically, ctDNA clearance occurred in 11% of the chemotherapy group vs 43% of the surveillance group. This analysis was based on only 16 ctDNA-positive patients (7 in surveillance, 9 in chemotherapy), highlighting the limited statistical power and the need for cautious interpretation. These findings underscore critical challenges in assay performance, patient selection, and endpoint choice that must be addressed in future studies. Despite these challenges, enrollment in well-designed prospective clinical trials remains the most effective strategy for advancing MRD-directed therapeutic approaches and establishing an evidence-based standard of care. It provides patients with access to novel therapies and accelerates the generation of high-quality evidence needed to establish a future standard of care for MRD-positive disease.

A systematic evaluation of the COBRA study failure highlights three contributing factors. First, patient selection likely introduced bias. The trial enrolled only low-risk stage II colon cancer patients, with a baseline recurrence risk below 15%.^42^ In such a low-event population, even an effective ctDNA-guided strategy is unlikely to show a clear benefit, as most patients are already cured by surgery alone. Second, limited assay sensitivity may have contributed to the failure. The tumor-informed platform used in COBRA was validated in high-risk cohorts. However, it may not have sufficient sensitivity to detect ultra-low ctDNA levels typically seen in low-risk stage II disease.^43^ Third, and notably, the trial relied on a single time-point endpoint—ctDNA clearance at 6 months, which proved misleading. Only 11% of patients receiving chemotherapy achieved ctDNA clearance, compared with 43% in the surveillance group.^42^ The most plausible explanation is the transient suppression of ctDNA shedding rather than the true eradication of residual disease. A single post-treatment measurement cannot distinguish temporary clearance from sustained molecular remission.

Managing an MRD-positive patient is further complicated by the timing of ctDNA detection. The critical meaning of a positive test result and the appropriate response are substantially influenced by the time of its detection. Current clinical protocols and trials are investigating several key time points.^6^ A postoperative landmark test, typically performed approximately 6 weeks after surgery, identifies patients with residual disease who are at the highest risk of recurrence. These patients are most likely to benefit from early initiation of adjuvant therapy.^44^ In contrast, longitudinal monitoring involves serial testing during and after the completion of adjuvant chemotherapy. Conversion from MRD-negative to MRD-positive during surveillance indicates molecular relapse. This finding should prompt radiologic evaluation and consideration of early intervention. A particularly challenging situation is persistent MRD positivity after completion of adjuvant chemotherapy. This suggests treatment failure and resistance to standard agents. In this setting, clinical trial enrollment becomes critical, as conventional options are limited. Thus, the interpretation of MRD status is closely linked to its timing. This temporal dimension adds considerable practical complexity to clinical decision-making [Figure 2].

**Figure 2:**
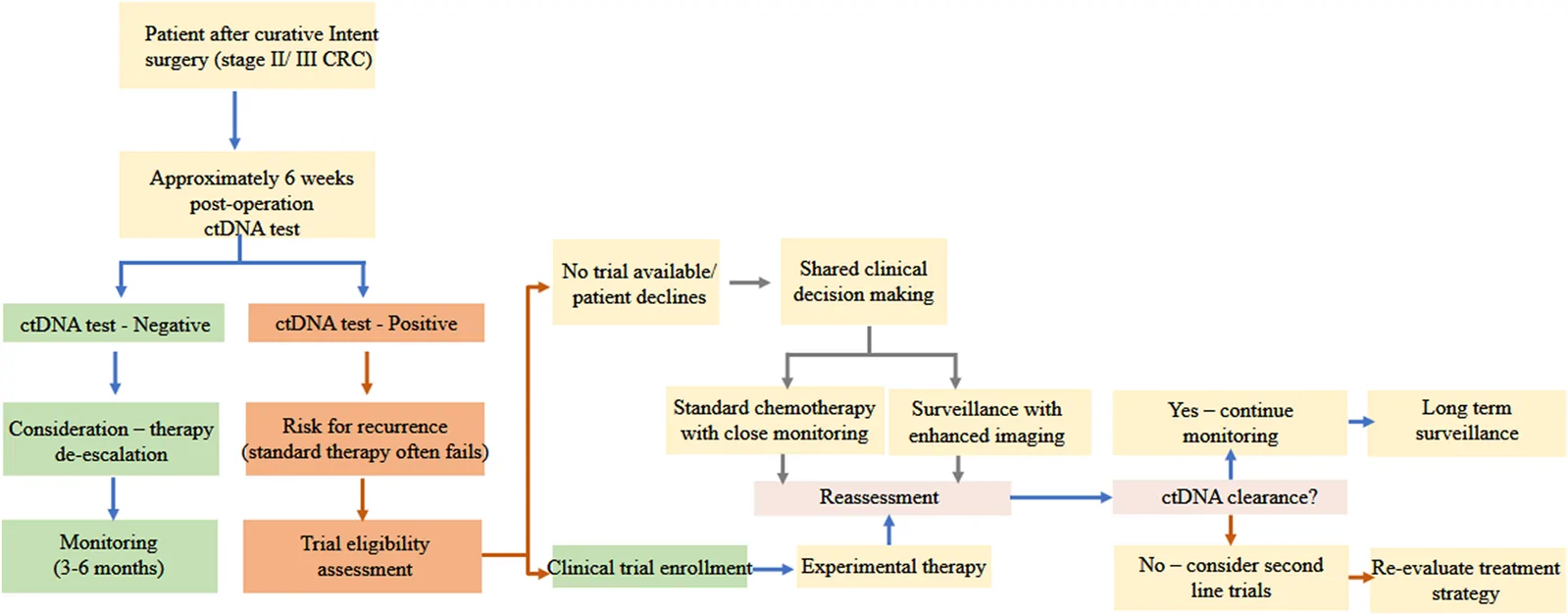
A proposed clinical decision pathway for the postoperative management of patients with stage II/III CRC after curative-intent surgery, incorporating ctDNA testing. The pathway begins with a ctDNA test approximately 6 weeks after surgery. A negative result leads to consideration of therapy de-escalation and long-term surveillance. A positive result indicates a high risk for recurrence, prompting a decision between standard chemotherapy, enhanced surveillance, or clinical trial enrollment if eligible. Subsequent decisions are based on ctDNA clearance and response to therapy. CRC: Colorectal cancer; ctDNA: Circulating tumor DNA; MRD: Minimal residual disease.

Based on prior evidences, future MRD-directed trials should incorporate three design improvements. First, studies should focus on higher-risk populations, such as stage III or high-risk stage II colon cancer. This approach increases event rates and improves statistical power, as demonstrated in recent ctDNA-guided studies and ongoing platform trials.^27^ Second, serial ctDNA monitoring should replace a single time-point assessment. Testing every 4–8 weeks during and after therapy can better capture ctDNA dynamics.^45^ A true molecular response should be defined as sustained ctDNA negativity at two or more consecutive time points. Third, assay performance must be validated in the intended clinical setting. The limit of detection should be below 0.01% variant allele frequency, as demonstrated in next-generation tumor-informed assays.^10^ Sensitivity must also be confirmed in low-shedding disease, where false-negative results remain a concern.^11^ Together, these refinements may reduce misclassification, improve trial accuracy, and support the development of effective MRD-directed therapies.

## The emerging evidence: categories of active investigation

The clear inadequacy of standard adjuvant chemotherapy for MRD-positive patients has prompted a global research effort to develop more effective intervention strategies. This shift reflects a decisive change away from uniform cytotoxic regimens toward a more nuanced, biologically informed approach in which therapeutic intensity is aligned with molecular risk. The following section outlines the main categories of ongoing clinical investigation. It highlights the scientific rationale and key trial designs that are currently being evaluated to address this critical unmet need [Figure 1 and Table 1].

**Table 1:** A comprehensive overview of interventional and observational trials evaluating therapeutic strategies informed by MRD status or targeting specific mechanisms of resistance in colorectal cancer.

Trial identifier	Trial name	Phase	Patient population	Intervention category	Experimental arm	Primary endpoint	Primary completion date
NCT04264702	BESPOKE CRC	II	Stage I-IV CRC, postoperative	Observational cohort	ctDNA-guided management	Clinical utility: Treatment decisions and recurrence outcomes	December 2025
NCT05174169	CIRCULATE-US (NRG-GI008)	II/III	High-risk stage II/III colon cancer, post-op ctDNA negative (de-escalation)	De-escalation	Standard chemotherapy vs. ctDNA-guided surveillance (de-escalation)	RFS (non-inferiority)	March 2030
NCT04486378	DYNAMIC-III	II/III	Stage III colon cancer, postoperative	ctDNA-guided therapy	ctDNA-guided adjuvant chemotherapy vs. standard management	RFS	August 2030
NCT07227636	–	II	Phase II, MSS CRC, post-op ctDNA+	Immunotherapy combination	BOT + BAL	ctDNA clearance at 6 months; RFS at 1 year	November 2030
NCT05571293	NEST-1	II	Resectable MSS/pMMR colorectal cancer	Immunotherapy (dual immune checkpoint blockade; neoadjuvant)	BOT + BAL, prior to surgery	MPR; ctDNA clearance and safety	July, 2026
ACTRN12617001560381	AGITG DYNAMIC-rectal	II/III	LARC, post-neoadjuvant chemoradiation, and TME	De-escalation/ctDNA-guided management	ctDNA-guided management vs. standard management (clinician's choice)	RFS	Completed in 2023
NRG-GI005/NCT04068103	COBRA	II/III	Resected low-risk stage II colon cancer	ctDNA-guided therapy (terminated)	Surveillance vs. FOLFOX/CAPOX if ctDNA+	ctDNA clearance at 6 months	Terminated (phase II)^a^
NCT05343013	INTERCEPT-TT trial	II	CRC with ctDNA-positive MRD after completing CIT (must include ≥3 months of oxaliplatin)	Cytotoxic therapy	TAS-102 (trifluridine/tipiracil) ± celecoxib	6-month ctDNA clearance rate	May 2027
NCT05534087	CLAUDIA colon cancer	II/III	Stage II-III colon cancer, post-op ctDNA+	Cytotoxic intensification	mFOLFIRINOX for 3 months following standard FOLFOX/CAPOX	3-year DFS	September 2030
NCT05018182	FOLFOXIRI neoadjuvant	II	High-risk locally advanced colon cancer	Cytotoxic intensification	Neoadjuvant FOLFOXIRI	TRG 0–2 rate	April 2022 (estimated) (status unknown)
NCT01640405	VISNU-1	III	mCRC with ≥3 CTCs, first-line	Cytotoxic intensification	FOLFOXIRI + Bevacizumab	PFS	Completed in 2018
NCT04607421	BREAKWATER	III	*BRAF* V600E-mutant mCRC, first-line	Targeted therapy	Encorafenib + cetuximab ± chemo	PFS, ORR	December 2027
NCT03384940	DESTINY-CRC01	II	*HER2*-expressing, RAS wild-type (mCRC) that progressed after ≥2 prior regimens	Targeted therapy (ADC)	Trastuzumab deruxtecan (T-DXd)	Confirmed ORR in cohort A	Completed in 2020
NCT04895137	BASKETⅡ	II	Advanced solid tumors, including CRC	Immunotherapy combination	Cohort A: mFOLFOX6 + Bevacizumab + nivolumab (CRC); Other cohorts: Dostarlimab + other agents	ORR per RECIST v1.1	Completed in 2024
NCT02997228	COMMIT (NRG-GI004)	III	First-line dMMR/MSI-H metastatic CRC	Immunotherapy combination	mFOLFOX6 + Bevacizumab + Atezolizumab vs. Atezolizumab monotherapy	PFS	June, 2027
NCT05999812	ATRA + Bevacizumab + Atezolizumab in MSS mCRC	II	Refractory MSS metastatic CRC, ≥2 prior lines	Immunotherapy combination	ATRA + Atezolizumab + Bevacizumab	ORR	October 2028
NCT06358430	–	I	CRC with MRD following complete disease resection	Cellular immunotherapy/targeted immunotherapy	TROP2-CAR-NK cells + cetuximab (with lymphodepleting chemotherapy: Fludarabine + Cyclophosphamide)	Safety, tolerability, MTD, and recommended phase II dose	January 2029
NCT03104439	–	II	Metastatic MSS CRC and pancreatic cancer	Immunotherapy + radiotherapy combination	Nivolumab + Ipilimumab + radiation in MSS CRC/PDAC	Disease control rate	April 2027
NCT04068610	COLUMBIA 1	Ib/II	Treatment-naïve metastatic MSS CRC	Immunotherapy combination	Durvalumab (anti-PD-L1) + Oleclumab (anti-CD73) + FOLFOX + Bevacizumab	Safety (phase Ib); ORR (phase II)	November 2026

Trials are categorized by intervention type, including immunotherapy combinations, cytotoxic intensification, targeted therapy, and ctDNA-guided management (de-escalation or escalation). Endpoints span feasibility, ctDNA clearance, RFS, and ORR, with completion dates ranging from 2018 to 2030. All NCT numbers link directly to registration records on https://clinicaltrials.gov/; ACTRN12617001560381 is available on the Australian New Zealand Clinical Trials Registry (ANZCTR): www.anzctr.org.au/. Ab: Antibody; ACTRN: Australian Clinical Trials Registry Number; ADC: Antibody–drug conjugate; BAL: Balstilimab; BOT: Botensilimab; CAPOX: Capecitabine + oxaliplatin; CAR: Chimeric antigen receptor; CIT: Cytotoxic intensification therapy; CRC: Colorectal cancer; CTC: Circulating tumor cell; ctDNA: Circulating tumor DNA; DFS: Disease-free survival; dMMR: Mismatch repair-deficient; Fc: Fragment crystallizable; FOLFOX: Folinic acid + fluorouracil + oxaliplatin; FOLFOXIRI: Folinic acid + fluorouracil + oxaliplatin + irinotecan; HER2: Human epidermal growth factor receptor 2; LARC: Locally advanced rectal cancer; mCRC: Metastatic colorectal cancer; MDSC: Myeloid-derived suppressor cell; mFOLFIRINOX: Modified folinic acid + fluorouracil + irinotecan + oxaliplatin; mFOLFOX6: Modified folinic acid + fluorouracil + oxaliplatin; MPR: Major pathological response; MRD: Minimal residual disease; MSI-H: Microsatellite instability-high; MSS: Microsatellite-stable; MTD: Maximum tolerated dose; NCT: National clinical trial; NK: Natural killer; ORR: Objective response rate; PD-1: Programmed cell death protein 1; PDAC: Pancreatic ductal adenocarcinoma; PD-L1: Programmed death-ligand 1; PFS: Progression-free survival; RFS: Recurrence-free survival; T-DXd: Trastuzumab deruxtecan; TME: Total mesorectal excision; Treg: Regulatory T cell; TRG: Tumor regression grade.

a Although the registry lists a primary completion date of June 2026, the phase II component was halted early after a prespecified futility analysis.

### Intensification of cytotoxic therapy

A direct, though unproven, hypothesis is that the failure of standard doublet chemotherapy (e.g., FOLFOX/CAPOX) to eradicate MRD might be overcome by intensifying treatment with multi-agent cytotoxic regimens. The rationale is that micrometastatic disease, although molecularly detectable, may still retain sufficient chemosensitivity when exposed to a broader spectrum of cytotoxic agents at higher doses and with diverse mechanisms of action. This approach also tests the hypothesis that dormant, chemoresistant CSCs thought to drive MRD persistence can be forced into the cell cycle and eliminated through a multi-mechanistic cytotoxic effect. This concept is being evaluated across multiple clinical settings, including neoadjuvant, first-line metastatic, and adjuvant MRD-positive settings.^12^^,^^46^, ^47^, ^48^, ^49^ Key studies include the phase II trial of FOLFOXIRI in high-risk locally advanced colon cancer (NCT05018182); the phase III VISNU-1 trial, which evaluates FOLFOXIRI plus bevacizumab in metastatic CRC (NCT01640405); and the phase II BREAKWATER study of encorafenib plus cetuximab with or without chemotherapy in *BRAF* V600E-mutant mCRC (NCT04607421).

A summary of representative trials, including estimated primary completion dates, is provided in Table 1. For example, NCT05018182 is a phase II neoadjuvant trial that evaluates modified FOLFIRINOX (5-FU, leucovorin, irinotecan, and oxaliplatin) in patients with high-risk locally advanced colon cancer before surgical resection. The trial is designed to determine whether intensified preoperative chemotherapy can improve pathological response and enhance long-term oncologic outcomes, including recurrence-free survival (RFS). Its anticipated completion may provide important evidence regarding the role of cytotoxic intensification in biologically high-risk localized disease.^49^ In postoperative settings, the multicenter platform CLAUDIA phase II/III trial (NCT05534087) uses tumor-informed postoperative ctDNA analysis to identify patients after curative resection for stage II–III colon cancer.^12^ MRD-positive patients are randomized upfront to receive either standard adjuvant chemotherapy (FOLFOX or CAPOX) or intensified modified FOLFIRINOX, with 3-year disease-free survival as the primary endpoint. This design directly tests whether MRD-guided treatment escalation improves outcomes. Early data demonstrate the feasibility of identifying and enrolling MRD-positive patients into intensified treatment strategies across multiple centers.^12^ However, these findings also highlight the challenge of balancing potential benefit with increased toxicity and underscore the need for randomized evidence to confirm a survival advantage. Additional trials are exploring intensified fluoropyrimidine-oxaliplatin regimens, including combinations such as EC plus modified FOLFOX-6 (mFOLFOX6) and triplet regimens like mFOLFOXIRI.^49^ In parallel, ctDNA-guided immunotherapy trials (e.g., NCT03832569) are evaluating pembrolizumab in high-risk, MRD-positive solid tumors, with ctDNA clearance and RFS as key efficacy endpoints. Whether cytotoxic intensification alone is sufficient remains uncertain.^50^ Ongoing studies will help determine if escalating chemotherapy intensity can effectively improve outcomes in the MRD-positive cases or if therapies with different mechanisms, such as immune checkpoint inhibitors, are ultimately required.

However, these intensified regimens are associated with substantially greater toxicity than standard doublet chemotherapy. Modified FOLFIRINOX, in particular, carries high rates of grade 3–4 hematologic and gastrointestinal events, including neutropenia, febrile neutropenia, and diarrhea. These toxicities often require dose reductions or the use of growth factor support.^51^ The phase II FROST trial (NCT05179889) is specifically evaluating the balance between safety and efficacy of adjuvant mFOLFIRINOX vs mFOLFOX6 in patients with high-risk stage III colon cancer, with treatment-related toxicity as a key secondary endpoint.^52^ Given these risks, careful patient selection is essential. Factors such as performance status, age, and organ function should guide the use of intensified regimens to minimize harm while preserving potential benefit.

### Integrating novel targeted agents

The established efficacy of targeted therapies in metastatic CRC provides a rationale for their evaluation in the ctDNA-positive MRD adjuvant setting. In this context, ctDNA serves as a dynamic biomarker to identify patients for molecularly matched targeted therapy aimed at eradicating micrometastatic disease driven by specific oncogenic alterations. This strategy aligns with the clonal evolution model, in which residual post-treatment clones may retain actionable vulnerabilities.^53^ This approach is particularly relevant for biomarker-defined CRC subtypes with established sensitivity in the metastatic setting. For example, in *BRAF* V600E-mutated CRC, ongoing studies are exploring *BRAF/*epidermal growth factor receptor (*EGFR*) inhibition in earlier-stage settings. The SWOG S2107 trial (NCT05308446) is a phase II study evaluating encorafenib plus cetuximab with or without nivolumab in pretreated, MSS, metastatic or unresectable *BRAF* V600E CRC, although it does not include ctDNA or MRD-based endpoints. In parallel, adjuvant trials incorporating ctDNA dynamics are investigating targeted agents, including *BRAF* inhibitors, in MRD-positive disease.^54^ Similarly, *HER2*-amplified CRC represents another promising subgroup. In the DESTINY-CRC01 trial (NCT03384940), the antibody–drug conjugate trastuzumab deruxtecan (T-DXd) demonstrated substantial activity in heavily pretreated metastatic CRC.^53^ These results support its potential role in MRD eradication, and adjuvant trials incorporating this agent are under development.^55^ The low tumor burden characteristic of MRD may increase sensitivity to targeted therapies, potentially improving clearance rates compared with chemotherapy alone. Results from ongoing adjuvant targeted therapy trials, as well as large MRD-focused platforms such as CIRCULATE and PRECISION-MRD (NCT06878131), are anticipated between 2027 and 2030, depending on enrollment and follow-up.^37^

### Immunotherapy for microsatellite-stable (MSS) cancers: overcoming the cold tumor microenvironment

The majority of CRCs are MSS and have been largely refractory to immune checkpoint inhibitors (ICIs) as monotherapy in the metastatic setting. However, the MRD-positive state presents a unique immunological context. The low tumor burden and potentially less immunosuppressive microenvironment of micrometastatic disease may be more susceptible to immune-mediated clearance. This provides a strong rationale for therapeutic strategies that combine T-cell activation with modulation of the tumor microenvironment to overcome immune resistance.

Single-agent checkpoint inhibition is being explored in dedicated MRD-positive populations, particularly in localized mismatch repair-deficient (dMMR)/microsatellite instability-high (MSI-H) malignancies. For example, the phase II NCT04082572 trial is assessing the neoadjuvant pembrolizumab in patients with localized dMMR/MSI-H solid tumors, with serial ctDNA analysis incorporated as an exploratory biomarker of treatment response. The primary findings indicate potential long-term clinical efficacy, while ctDNA clearance following pembrolizumab was strongly associated with pathological complete response and improved event-free and overall survival.^56^^,^^57^ With completion expected in December 2028, this study will help define the role of single-agent ICI in the MRD setting.

Combination strategies with vascular modulators are also under investigation. In MSI-H/dMMR metastatic CRC, the CheckMate 142 trial (NCT02060188) established the efficacy of nivolumab with or without ipilimumab.^58^ For more common MSS settings, trials such as NCT05999812 are evaluating ICIs in combination with vascular endothelial growth factor (VEGF) inhibitors (e.g., atezolizumab + bevacizumab). These approaches are based on the concept that normalization of tumor vasculature and modulation of the immune microenvironment may enhance T-cell infiltration into the micrometastatic disease.^58^

Next-generation immunotherapy combinations designed to overcome the immune resistance of MSS CRC are emerging. A notable example is the combination of botensilimab (BOT), an Fc-enhanced anti-cytotoxic T-lymphocyte-associated protein 4 (CTLA-4) antibody, and balstilimab (BAL), an anti-programmed cell death protein 1 (anti-PD-1) antibody. This dual checkpoint inhibition strategy is based on Fc engineering of the anti-CTLA-4 component, which enhances depletion of intratumoral regulatory T cells and promotes a robust antitumor immune response in immunologically ‘cold’ tumors. Early-phase studies in refractory metastatic MSS CRC have shown encouraging activity, particularly in patients without active liver metastases, with objective response rates of approximately 27.6% and durable disease control.^59^^,^^60^ Notably, neoadjuvant BOT/BAL in the NEST-1 trial (NCT05571293) induced deep pathological responses in locally advanced MSS CRC and resulted in sustained ctDNA negativity.^61^ Building on this,a dedicated phase II trial (NCT07227636) is now evaluating this combination in patients with MSS CRC who remain ctDNA-positive after curative-intent surgery and adjuvant chemotherapy.^62^

Immunotherapy in MSS CRC carries significant risks. In a phase I study of BOT + BAL, 89.0% of patients experienced treatment-related adverse events, most commonly fatigue, diarrhea, and pyrexia, and 12.0% discontinued therapy due to toxicity.^60^ Immune-related toxicities such as colitis, hepatitis, and endocrinopathies are relevant considerations. These risks, along with potential toxicity from combination strategies, underscore the need for careful patient selection and close monitoring in MRD-directed trials.

Additional strategies aim to target the immunosuppressive tumor microenvironment. One example is the combination of durvalumab, an anti-programmed death-ligand 1 (PD-L1), and oleclumab (anti-CD73), currently being evaluated in the phase Ib/II COLUMBIA-1 trial (NCT04068610) therapy in combination with chemotherapy and bevacizumab. By inhibiting CD73, this approach aims to reduce immunosuppression and enhance T-cell activity against micrometastatic disease.^63^

Radiotherapy-immunotherapy combinations also represent a promising approach. Radiation can induce immunogenic cell death, increase the release of tumor-associated antigens, and remodel the tumor microenvironment, potentially converting immunologically cold tumors into hot tumors that are more responsive to ICIs. The phase II trial (NCT03104439) is evaluating stereotactic body radiotherapy (SBRT) combined with ipilimumab plus nivolumab in patients with metastatic MSS CRC. In the intention-to-treat population, the study was designed to evaluate efficacy, and ctDNA dynamics were explored as a correlative biomarker.^64^

Dual immune checkpoint blockade targeting alternative inhibitory receptors beyond PD-1/PD-L1 is also under investigation. The LAG-3 inhibitor, relatlimab, combined with nivolumab, was evaluated in patients with previously treated metastatic proficient mismatch repair (pMMR) and MSS CRC in a phase II trial (NCT03642067).^65^ Although the study did not meet its primary endpoint, it provided insights into the effects of LAG-3/PD-1 blockade on the tumor microenvironment and identified a subgroup of patients with lung-only metastases. Preclinical data suggest that these alternative checkpoint pathways are upregulated in ICI-resistant tumors and may represent compensatory immune escape mechanisms.

Metabolic reprogramming combined with ICIs targets the distinct metabolic landscape of the tumor microenvironment. Arginine metabolism, adenosine signaling, and glutamine blockade are all being explored. The adenosine pathway inhibitor oleclumab (anti-CD73) was evaluated in combination with durvalumab and chemotherapy in the phase Ib/II COLUMBIA-1 trial (NCT04068610) in patients with treatment-naïve metastatic MSS CRC.^63^ Although the combination has shown a manageable safety profile, its efficacy has not supported further development in MSS CRC. The arginase inhibitor INCB001158 has been tested with pembrolizumab in advanced solid tumors, including MSS CRC (NCT02903914), but objective response rates did not exceed background activity for these tumor types.^66^

Novel immune agonists activate innate immunity to prime adaptive responses. Stimulator of interferon genes (STING) agonists like ADU-S100 (MIW815) promote type I interferon production and dendritic cell activation. The phase I trial NCT02675439 demonstrated target engagement and a manageable safety profile in advanced solid tumors, though antitumor activity remained limited.^67^ Preclinical colon carcinoma models suggest STING agonists may synergize with chemotherapy or toll-like receptor 9 (TLR9) agonists.^68^ TLR9 agonists, such as SD-101, have demonstrated synergy with PD-1 blockade in preclinical colon cancer models and are under investigation in solid tumors (NCT02521870), although no active CRC MRD-specific trials have been identified.

Nevertheless, it is important to note that these approaches are experimental, and the current evidence base is limited to trial design and early phase feasibility, rather than definitive clinical outcomes. The strategies described above illustrate the growing array of tools available for addressing MSS CRC immune resistance and are being increasingly incorporated into MRD-centric trials. Their ultimate clinical utility will depend on rigorous prospective validation in well-designed randomized studies.

### De-escalation in minimal residual disease (MRD)-negative patients

A complete view of the MRD paradigm also requires recognizing its counterpart, namely, using MRD negativity to safely de-escalate therapy. In this context, the DYNAMIC trial provided level 1 evidence that omitting adjuvant chemotherapy in ctDNA-negative stage II colon cancer was non-inferior to standard management and substantially reduced the number of patients exposed to unnecessary treatment toxicity.^26^ This principle is now being systematically extended beyond the initial studies to larger programs. Large-scale platform trials, such as the CIRCULATE-US trial (NCT05174169), also known as NRG-GI008, are actively randomizing ctDNA-negative patients with stage II and stage III disease either to receive standard adjuvant chemotherapy or de-escalated therapy/observation.^69^ With a primary completion date estimated for 2030, this and similar trials will ultimately define the standard of care for low-risk patients.

The MRD-guided de-escalation paradigm has been extended to rectal cancer through the phase II AGITG DYNAMIC-Rectal trial (ACTRN12617001560381).^70^ Eligible patients with locally advanced rectal cancer received neoadjuvant chemoradiation and were randomized in a 2:1 ratio to ctDNA-informed vs standard adjuvant management prior to total mesorectal excision. In the experimental arm, patients who were ctDNA-negative at both weeks 4 and 7 post-surgery and pathologically node-negative avoided adjuvant chemotherapy, whereas ctDNA-positive or node-positive patients received standard therapy.^70^

The results demonstrated that a ctDNA-guided strategy significantly reduced the use of adjuvant chemotherapy (46% vs. 77%; *P* < 0.001). Interestingly, the use of oxaliplatin-based doublets was similar between the groups (28% vs. 25%). The 3-year RFS rate was 76% in the ctDNA-guided group and 82% in the standard management group (difference 6%, 95% CI: −6%–17%), suggesting that the trial was underpowered to draw definitive conclusions about oncologic efficacy, although it demonstrated the feasibility of ctDNA-guided de-escalation. However, the small sample size precludes definitive conclusions regarding non-inferiority in RFS. The trial also highlighted key limitations of ctDNA detection. Among patients who remained ctDNA-negative but experienced recurrence, 80% had lung-only metastases.^70^ This suggests that pulmonary lesions may shed insufficient ctDNA for detection with current assays. These findings underscore the ongoing need for refinement of MRD technologies and the integration of multiple parameters, including ctDNA status, pathology, and radiographic monitoring, into comprehensive de-escalation strategies.

The data from these studies will be important in validating the ctDNA-negative status as a robust biomarker for selecting patients who can safely avoid chemotherapy. This will thereby complete the vision of a fully personalized adjuvant strategy in which intervention is dynamically calibrated to individual molecular risk [Figure 2].

### Future directions in minimal residual disease (MRD)-directed immunotherapy

Despite growing progress, effective immunotherapy for MSS CRC remains limited. This has prompted the exploration of several emerging strategies aimed at overcoming immune resistance in the MRD setting.

Innate immune activation represents a key area of investigation. Agents such as STING agonists and TLR9 agonists are designed to enhance antigen presentation and induce type I interferon signaling. Early-phase studies have demonstrated biological activity, including immune priming and modulation of the tumor microenvironment. However, clinical efficacy in CRC remains modest, and their role in MRD-directed therapy is not yet established. STING agonists (e.g., ADU-S100/MIW815) have shown limited single-agent activity but clear immunologic engagement in early phase I trials, particularly in combination with checkpoint blockade.^71^ Cellular immunotherapies are also under active development. Approaches such as chimeric antigen receptor natural killer (CAR-NK) cells and other adoptive immune strategies aim to directly target residual tumor cells. These therapies offer theoretical advantages in low-volume disease, including enhanced specificity and reduced tumor heterogeneity.^72^^,^^73^ Nonetheless, current evidence is confined to early-phase trials, and significant challenges remain, including persistence, trafficking, and scalability.

Targeting the metabolic and immunosuppressive tumor microenvironment represents another promising avenue of investigation. Strategies focused on adenosine signaling, arginine metabolism, and related pathways aim to reverse immune suppression and restore effective antitumor responses.^74^^,^^75^ Emerging evidence indicates that metabolic reprogramming and reciprocal interactions between tumor cells and the surrounding microenvironment create therapy-resistant niches that facilitate immune escape and disease persistence. Consequently, nanotechnology-enabled therapeutic platforms capable of simultaneously modulating the tumor microenvironment and improving drug delivery have emerged as promising adjuncts to immunotherapy in precision oncology.^76^ While preclinical data are encouraging, clinical translation has been inconsistent, with limited efficacy observed in early trials. Additional investigational approaches include novel immune agonists and combination strategies, designed to enhance both innate and adaptive immunity. These include agents targeting alternative checkpoint pathways such as LAG-3, T-cell immunoreceptor with Ig and ITIM domains (TIGIT), and TIM-3, as well as rational combinations integrating innate immune stimulators with PD-1/PD-L1 blockade.^77^ Early-phase studies suggest potential synergy in immune activation and tumor microenvironment remodeling, but these approaches remain exploratory and lack robust clinical validation in MRD-directed CRC therapy. Overall, these emerging strategies highlight the extent of innovation in MRD-directed immunotherapy.

## The path forward: validation and implementation

The integration of ctDNA-based MRD detection into routine clinical management requires overcoming two major challenges. First, ctDNA must be formally validated as a surrogate endpoint for drug development, a process that remains a key bottleneck. Second, practical barriers to the widespread and equitable implementation of this approach must be addressed.

### ctDNA as a surrogate endpoint: the data so far

The extended timeline required to assess overall survival (OS) in the adjuvant setting, often exceeding a decade, presents a major barrier to the development of new therapies. The strong association between ctDNA clearance and clinical outcomes has positioned ctDNA dynamics as a potential surrogate endpoint to accelerate trial evaluation. A growing body of evidence supports the use of ctDNA dynamics as an indicator of therapeutic response. A meta-analysis of patients with early-stage CRC demonstrated that postoperative ctDNA detection following surgery was the strongest predictor of recurrence, whereas ctDNA clearance following adjuvant chemotherapy was associated with significantly improved RFS.^78^ These findings highlight the prognostic value of ctDNA dynamics as an early indicator of treatment response.

Nevertheless, ctDNA clearance should be interpreted with appropriate caution, as apparent molecular clearance may not always reflect complete eradication of MRD. For example, findings from the COBRA trial indicate that chemotherapy may transiently suppress ctDNA shedding rather than eliminate residual tumor cells, emphasizing the need to interpret ctDNA kinetics within the broader clinical context and to validate molecular clearance using long-term clinical outcomes. Subsequent prospective studies in CRC and other solid tumors have consistently replicated the finding that post-treatment ctDNA clearance correlates with superior RFS.^29^^,^^79^ Recognizing this potential, regulatory agencies and international consortia have initiated large-scale efforts to validate ctDNA as a surrogate endpoint. This work has been further formalized in a recent consensus report from the National Cancer Institute ctDNA Gastrointestinal (NCI GI) Working Group, which outlines a framework for the clinical and regulatory validation. The report emphasizes the need for standardized assay performance, harmonized definitions of molecular response, and the use of ctDNA kinetics rather than single time-point measurements.^80^ Importantly, it highlights that validation must occur at both the individual patient level and the trial level, requiring large pooled analyses from randomized studies. These recommendations provide a clear roadmap for integrating ctDNA into drug development and are expected to play a key role in enabling regulatory acceptance of MRD-directed therapeutic strategies.

The U.S. Food and Drug Administration (FDA) and the NCI have established collaborative working groups, including the NCI Colon Cancer Task Force (FDA guidance page), which have outlined a clear pathway for validating ctDNA as a biomarker in CRC.^80^^,^^81^ These efforts build on the foundational work of large international consortia such as Adjuvant Colon Cancer End Points (ACCENT) and International Duration Evaluation of Adjuvant Chemotherapy (IDEA). Pooled analyses from these groups, involving more than 40,000 patients, established disease-free survival as a valid surrogate for OS and helped define modern adjuvant treatment standards.^82^ Recent analyses from the IDEA-France and HORG-IDEA-Greece cohorts show that postoperative ctDNA is the strongest independent prognostic factor for recurrence and survival in stage III colon cancer. These findings are increasingly influencing ongoing ACCENT-associated analytic work.^83^

The next critical step is the conduct of large, prospective meta-analyses that pool individual patient data from multiple adjuvant trials. These analyses are needed to rigorously define the relationship between ctDNA endpoints and long-term outcomes such as RFS and OS. This effort is being led by international consortia designed for this purpose. The ACCENT consortium, with data from more than 40,000 patients across prior trials, is now actively working to validate ctDNA endpoints through pooled analyses.^83^^,^^84^ In parallel, the International ctDNA Expert Panel & Standards (IDEAS) initiative is developing standardized definitions for ctDNA-based endpoints to ensure consistency across future trials.^84^

A key step in this process is defining the most appropriate ctDNA endpoint. Several candidate metrics are currently under evaluation, including ctDNA clearance, ctDNA molecular response, and undetectable ctDNA status. ctDNA clearance is currently defined as a conversion from a positive to a negative result at a predefined time point, such as 12–16 weeks after initiating adjuvant therapy. ctDNA molecular response refers to a defined relative reduction in ctDNA levels compared with baseline.^39^ In contrast, undetectable ctDNA at the end of treatment is considered a marker of complete molecular response.

The current debate centers on whether a binary ctDNA clearance endpoint or a continuous measure of ctDNA kinetics better correlates with long-term survival and is more practical for clinical trials. This issue was highlighted by the COBRA trial, which used ctDNA clearance at a single time point as its primary endpoint.^43^ The unexpected finding of lower clearance rates in patients receiving chemotherapy (11%) compared with those under surveillance (43%) suggests that single-time-point clearance may reflect temporary suppression rather than true eradication of disease. This underscores the need for more robust validation of ctDNA endpoints as surrogates for long-term outcomes. Validation of these endpoints could substantially accelerate the approval of new adjuvant therapies and reshape clinical trial design in CRC.

From a regulatory perspective, the U.S. FDA recognizes two distinct categories of surrogate endpoints: validated surrogates and those considered “reasonably likely” to predict clinical benefit.^85^ Validated surrogate endpoints, such as disease-free survival in CRC, show strong and consistent correlation with OS and can support traditional drug approval. In contrast, endpoints that are reasonably likely to predict clinical benefit may be used under the Accelerated Approval pathway (https://www.fda.gov/drugs/nda-and-bla-approvals/accelerated-approval-program) contingent upon subsequent confirmatory trials to verify clinical benefit.

For ctDNA-based MRD endpoints, current evidence supports strong prognostic value, but their full validation as surrogate endpoints for survival outcomes has not yet been achieved. Therefore, ctDNA clearance and molecular response are best considered as endpoints that are reasonably likely to predict clinical benefit. Regulatory guidance emphasizes that acceptance of such endpoints requires consistent evidence across multiple randomized trials. This includes demonstrating that treatment effects are reflected in both the surrogate endpoint and clinical outcomes, along with clear biological plausibility and standardized assay performance.^86^ Ongoing efforts by international consortia and cooperative groups aim to generate this level of evidence. Successful validation of ctDNA dynamics as a surrogate endpoint would accelerate drug development while maintaining rigorous standards for clinical benefit.

### Implementation challenges and clinical guidance

Beyond biological validation, the integration of MRD testing into routine oncology practice faces significant practical obstacles documented in health services research.

#### Access, equity, and cost-effectiveness

The availability of advanced ctDNA testing remains variable across healthcare systems, raising substantial concerns about equity in cancer care. The high cost of testing, inconsistent insurance coverage, and dependence on centralized testing laboratories limit the accessibility and implementation of ctDNA assays. These barriers disproportionately affect underserved communities, including patients from underrepresented racial and ethnic minority groups, those in rural regions, and patients receiving care within resource-limited healthcare systems.^87^ Emerging initiatives, including multi-cancer early detection (MCED) programs, aim to expand access through sponsored testing initiatives and targeted implementation strategies for underserved populations.^87^^,^^88^ However, these efforts remain in the early stages of development, and effectiveness in improving real-world access and reducing healthcare disparities has not yet been established. Additionally, several academic centers are developing in-house ctDNA assays and alternate analytical strategies to lower testing costs.^89^ Ensuring that these technological advances translate into equitable clinical access remains a healthcare priority for the broader implementation of ctDNA technologies.

The second major practical challenge to the widespread adoption of ctDNA-guided management is its cost-effectiveness. While MRD-guided strategies have the potential to reduce overtreatment, treatment-related toxicity, and overall healthcare utilization, they remain expensive. Further, reimbursement policies also remain inconsistent. In the United States, Medicare has established a national framework for selected ctDNA assays in advanced disease, whereas reimbursement for MRD testing in the adjuvant setting varies widely across private insurers and geographic regions. Early health economic models are beginning to address this issue. Initial models suggest that ctDNA-guided approaches may be cost-effective primarily by avoiding unnecessary chemotherapy in MRD-negative patients. However, these findings are highly sensitive to factors such as assay pricing, chemotherapy expenses, and healthcare system structure. Prospective health economic studies are therefore needed to refine these models and generate robust evidence of value to support broader reimbursement and clinical adoption.

#### Management of discordant results minimal residual disease-positive/radiology-negative (MRD+/RAD-)

The management of discordant results presents a significant clinical challenge, particularly in patients who are ctDNA-positive but lack radiographic evidence of disease (MRD+/RAD−). In this setting, the absence of detectable lesions on conventional imaging does not necessarily exclude residual disease, and additional imaging modalities are often required to identify hidden sites of occult metastases. Positron emission tomography/computed tomography (PET/CT) has demonstrated high diagnostic sensitivity for detecting occult disease in patients with biochemical recurrence despite negative conventional imaging, with more than half remaining eligible for curative-intent treatment.^90^ Magnetic resonance imaging (MRI) with diffusion-weighted sequences provides higher sensitivity for small lesions, particularly liver metastases and pelvic recurrences less than 10 mm that may be missed on CT.^91^ In female patients, dedicated pelvic MRI or transvaginal ultrasound is also important to exclude ovarian metastases (Krukenberg tumors), which are often bilateral and may be subtle or occult on CT imaging.^92^

In patients with mucinous carcinoma, signet-ring histology, or appendiceal primary tumors, diagnostic laparoscopy should be considered. This approach can detect radiographically occult peritoneal disease in approximately 20–30% of cases and thereby prevent unnecessary laparotomy.^93^^,^^94^ A systematic diagnostic evaluation facilitates the identification of patients with limited, resectable disease who may benefit from metastasectomy or other curative-intent investigations. It also minimizes the risk of inadvertent enrollment of patients with undetected macroscopic disease into trials intended for true MRD. If no visible disease is found after comprehensive evaluation, current guidelines recommend enrollment in clinical trials. To further support clinical decision making, several institutions are developing electronic health record-integrated clinical decision support tools that provide point-of-care guidance on MRD interpretation and management options.

#### False-negative minimal residual disease (MRD) results: a critical safety concern

While much attention has focused on false-positive MRD results due to clonal hematopoiesis, false-negative results are also important. False negatives can delay relapse detection and lead to undertreatment.^95^ ctDNA-based MRD assays are particularly prone to false negatives in specific settings. These include low tumor shedding, where ctDNA levels fall below the assay's limit of detection. False negatives may also occur when the selected mutation panel does not adequately capture the genomic profile of the recurrent clone.

***Low ctDNA shedding in peritoneal and pulmonary metastases:*** Low ctDNA shedding in certain metastatic sites presents a notable limitation of MRD detection. Tumor shedding varies widely, and the site of disease strongly influences ctDNA detectability. Peritoneal metastases, in particular, are often relatively isolated from the systemic circulation. As a result, they may produce very low plasma ctDNA levels despite considerable disease burden. Pulmonary metastases can also exhibit low shedding in some cases. These patterns raise concern that a negative plasma ctDNA result does not reliably exclude residual disease in such patients. Supporting this, isolated peritoneal metastases in CRC have consistently been associated with lower ctDNA detection rates compared with other metastatic sites.^95^

***Clonal hematopoiesis interference:*** Clonal hematopoiesis is primarily a source of false-positive ctDNA findings. However, it may also complicate MRD interpretation when assay design or variant selection is suboptimal.^10^^,^^95^ Sequencing tumor tissue paired with white blood cells improves identification of somatic variants and helps filter clonal hematopoiesis of indeterminate potential (CHIP)-related mutations. This approach can improve the reliability of tumor-informed assays. Omitting this step risks misinterpreting blood-derived mutations as cancer signals in plasma, which can disrupt MRD tracking.

***Synchronous multiple primary tumors:*** Tumor-informed assays are typically based on the genomic profile of a single index lesion. As a result, they may fail to detect recurrence or progression arising from a second primary tumor with a distinct molecular profile. A 2024 case report in synchronous CRC highlights this limitation: the MRD assay was initially negative and became informative only after it was redesigned using the alternate primary tumor. The authors concluded that single-tumor-informed approaches may miss both synchronous and metachronous disease.^96^ Similar findings have been reported in synchronous urothelial carcinoma and lung adenocarcinoma, in which ctDNA analysis revealed mutation patterns from multiple tumor lineages.^97^^,^^98^ These observations suggest that ctDNA can capture signals from coexisting primary tumors, even when those signals may not be represented in a single-tumor-informed design. In patients with synchronous cancers or suspected multifocal disease, broader plasma-only approaches could offer advantages. Methylation-based assays and multi-analyte liquid biopsy platforms may provide a more comprehensive assessment of total disease burden compared with single-tumor-informed assays.^99^

***Surveillance in low-shedding tumors:*** Surveillance is more challenging in low-shedding tumors, including peritoneal disease, lung metastases, mucinous histology, and other ctDNA-low phenotypes. In these settings, a negative ctDNA result should not be used in isolation to de-escalate follow-up. Expert guidance emphasizes that ctDNA-negative findings can be difficult to interpret and should be integrated with imaging and other biomarkers rather than being considered in isolation. In clinical practice, this approach supports continued radiographic surveillance and serial carcinoembryonic antigen (CEA) monitoring.^100^^,^^101^ In selected cases, particularly in patients with mucinous or appendiceal tumors and suspected occult peritoneal disease, additional evaluation with MRI, PET/CT, or diagnostic laparoscopy may be appropriate.^100^ Recurrence in a patient who remains ctDNA-negative highlights important limitations of current MRD assays. It raises questions about analytical sensitivity, tumor shedding biology, and platform performance. Variability in assay design, turnaround time, and reporting standards further complicates interpretation. These challenges emphasize the need for clear clinical algorithms, standardized reporting, and targeted clinician education.^102^ Emerging technologies may help address some of these gaps. Methylation-based assays are particularly promising, as cancer-specific DNA methylation patterns are abundant and can provide additional information on tissue of origin.^21^ This approach may enable simultaneous detection and localization of MRD. However, all current ctDNA platforms remain susceptible to interference from CHIP. These age-related mutations in hematopoietic cells release DNA into plasma and can lead to false-positive results if not appropriately filtered using matched white blood cell sequencing.^103^

Beyond the biological limitation described above, several technical factors can further reduce assay sensitivity. Consistent with findings from the DYNAMIC-Rectal trial, even extensive peritoneal carcinomatosis may remain undetectable in plasma, and patients with very low tumor burden can result in ctDNA levels near the limit of detection, leading to intermittent false-negative results.^70^^,^^104^ Gene-specific detection bias has been reported, with alterations in genes such as adenomatous polyposis coli (*APC*) detected less reliably than tumor protein p53 (*TP53*) or kirsten rat sarcoma viral oncogene homolog (*KRAS*) variants, possibly due to differences in DNA release or fragmentation patterns.^105^ Taken together, these biological and technical limitations support a multimodal surveillance strategy that integrates ctDNA analysis with serial imaging and other biomarkers. They also highlight the need for continued technological refinement to improve sensitivity, particularly in low-shedding tumors.

#### Clinical decision pathway and patient communication

For clinicians implementing MRD testing, several practical considerations are essential. First, the clinical context should guide assay selection. Tumor-informed assays are generally preferred in the adjuvant setting because of their high sensitivity, whereas plasma-only assays may be more appropriate for monitoring established metastatic disease. Second, time optimization is critical. Postoperative testing at 4–6 weeks provides the most validated prognostic information. Serial monitoring can detect molecular relapse earlier than conventional imaging [Figure 2]. Third, institutions should develop clear management pathways for interpreting and managing discordant results, particularly the MRD-positive/radiology-negative (MRD+/RAD-) scenario, in which clinical trial enrollment should be prioritized. Finally, patient communication is essential. MRD positivity indicates an increased risk of recurrence but does not guarantee relapse. It is also important to explain that current standard therapies have limited effectiveness in this setting, thereby making participation in clinical trials particularly important.

Professional societies, including the American Society of Clinical Oncology (ASCO) and the National Comprehensive Cancer Network (NCCN), are actively developing guidelines and educational programs to standardize the use of MRD testing in clinical practice.^106^ These efforts aim to clarify the appropriate use of ctDNA results in guiding treatment decisions, thereby enabling clinicians to integrate this evolving evidence into routine clinical practice safely and consistently.

### Emerging technologies and international coordination efforts

In addition to current ctDNA assays, several emerging technologies may further improve MRD detection. Fragmentomics, which examines ctDNA fragment size distribution, fragmentation patterns, and end motifs, is a particularly promising approach. It can detect cancer-specific ctDNA fragmentation signals without relying on tumor-specific mutations.^107^ Early clinical studies suggest that fragmentomic profiles can detect MRD with high accuracy in CRC, with reported sensitivities exceeding 90% in some cohorts.^108^ Advances in DNA methylation analysis also hold considerable promise. These assays target cancer-specific epigenetic changes, particularly at CpG sites, and may provide improved sensitivity and tumor-type specificity.^109^ In CRC, methylation-based MRD assays have demonstrated high clinical specificity (approximately 95%) and can detect recurrence several months before radiographic evidence of recurrence.^110^ Importantly, these approaches may enable highly sensitive MRD detection while simultaneously providing information on the tissue of origin. Despite these advances, their clinical implementation requires standardization. International harmonization efforts are underway to address this need. Initiatives such as the International Liquid Biopsy Standardization Alliance (ILSA) and consortia such as CANCER-ID aim to establish technical standards, validation frameworks, and proficiency testing programs to ensure consistent performance across platforms and across geographical regions.^111^^,^^112^ The successful adoption of these technologies will depend on both continued technical innovation and coordinated global collaborations.

## Study limitations

This review provides a comprehensive overview of the current landscape of MRD detection and therapeutic strategies in CRC. However, several points should be considered when interpreting the findings. The field is evolving rapidly, and some ongoing trials may have updated findings or undergone changes in status since the literature search. This review focuses mainly on ctDNA-based MRD strategies and does not comprehensively address other emerging biomarkers, such as circulating tumor cells and exosomes, biomarkers that may serve as complementary approaches for MRD detection. Although representative trials are discussed, this review does not include all geographically diverse or smaller-scale investigations. In addition, heterogeneity in ctDNA assay platforms, the timing of assessment, and definitions of MRD positivity across studies limits direct comparisons. Most therapies described remain investigational, and long-term survival is not yet available for many MRD-directed interventions. Despite these limitations, this review synthesizes the available evidence and provides a comprehensive framework for understanding emerging therapeutic strategies for MRD-positive CRC.

## Conclusion and prospects

The management of early-stage CRC is transitioning from a one-size-fits-all, pathology-based paradigm to a dynamic, risk-adapted strategy guided by individual molecular signatures. ctDNA-based MRD detection has emerged as a powerful prognostic biomarker that improves risk stratification and enables the safe de-escalation of adjuvant chemotherapy for patients cured by surgery alone. However, this progress also highlights a critical challenge: effective treatments for patients with high-risk MRD-positive disease remain limited. The current priorities are therefore twofold. First, we must identify and validate effective therapeutic strategies through clinical trials investigating intensified chemotherapy, MRD-guided targeted therapies, and novel immunotherapeutic combinations. Second, we must establish ctDNA clearance as a validated surrogate endpoint for regulatory drug approval.

Achieving these goals requires coordinated efforts across the clinical and research communities. Regulatory qualification of ctDNA-based MRD endpoints as surrogate markers of clinical benefit will be pivotal. Such a qualification has the potential to accelerate drug development in early-stage CRC. Successful enrollment of MRD-positive patients in clinical trials, coupled with the validation of ctDNA-based endpoints, will help translate the promise of liquid biopsy into tangible survival benefits. Ultimately, this approach will enable a truly personalized and effective adjuvant strategy for patients with CRC.

## Authors contribution

Yusuf Saleem Khan and Aisha Farhana conceptualized the review, designed the methodology, performed the primary literature analysis and synthesis, and created the visualizations. Abdullah Alsrhani, Hatim Adam Nagi, Alfatih Mohamed Ahmed Alnajib, Tarig Ginawi, Abuzar Abdulwahab Osman, and Emad Manni contributed to screening studies, data extraction, and the critical validation of the synthesized information, as well as reviewing and editing the manuscript. Yusuf Saleem Khan and Aisha Farhana wrote the original draft. All authors reviewed and approved the final version of the manuscript.

## Ethics statement

Not applicable.

## Data availability statement

The datasets used in the review are available from the corresponding author on reasonable request.

## Declaration of generative AI and AI-assisted technologies

The authors declare that no generative AI or AI-assisted technologies were used in the preparation of this manuscript.

## Funding

This research was funded by the 10.13039/501100023674Scientific Research Deanship at the University of Ha'il, Saudi Arabia through project number RG-25 012.

## Conflicts of interest

The authors declare that they have no known competing financial interests or personal relationships that could have appeared to influence the work reported in this paper.
